# Association of Plasma Branched-Chain and Aromatic Amino Acids with Reduction in Kidney Function Evaluated in Apparently Healthy Adults

**DOI:** 10.3390/jcm10225234

**Published:** 2021-11-10

**Authors:** MH Mahbub, Natsu Yamaguchi, Yuki Nakagami, Ryosuke Hase, Hidekazu Takahashi, Yasutaka Ishimaru, Rie Watanabe, Hiroyuki Saito, Junki Shimokawa, Hiroshi Yamamoto, Shinya Kikuchi, Tsuyoshi Tanabe

**Affiliations:** 1 Department of Public Health and Preventive Medicine, Yamaguchi University Graduate School of Medicine, Ube 755-8505, Japan; natsu@yamaguchi-u.ac.jp (N.Y.); nakagami@yamaguchi-u.ac.jp (Y.N.); hase@yamaguchi-u.ac.jp (R.H.); ishimaru.yasutaka@pref.yamaguchi.lg.jp (Y.I.); nell3wtnb@gmail.com (R.W.); hirosaitojapan@gmail.com (H.S.); junki.shimokawa.och49@gmail.com (J.S.); tanabe@yamaguchi-u.ac.jp (T.T.); 2 Department of Public Health, Faculty of Veterinary Medicine, Okayama University of Science, Imabari 794-8555, Japan; h-takahashi@vet.ous.ac.jp; 3 Institute for Innovation, Ajinomoto Co., Inc., Kawasaki 210-8681, Japan; hiroshiA_yamamoto@ajinomoto.com (H.Y.); shinya_kikuchi@ajinomoto.com (S.K.)

**Keywords:** plasma amino acids, branched-chain, aromatic, glomerular filtration rate, kidney function decline

## Abstract

The published literature on the association of circulatory branched-chain amino acids (BCAAs) and aromatic amino acids (AAAs) with reduced kidney function is inconsistent or conflicting. Clarification of it might help to better understand the underlying pathophysiology and to determine potential biomarkers for early detection and evaluation of kidney function decline. Our main purpose was to explore and clarify the potential relationships of individual BCAAs and AAAs with estimated glomerular filtration rate (eGFR) decline. We included the data from 2804 healthy subjects and categorized them into three groups based on eGFR tertiles. The associations between individual amino acids and eGFR were explored by covariate-adjusted logistic regression models. There was a progressive increase in the concentrations of BCAAs and AAAs from the upper to the lower tertiles. We revealed significant positive associations of isoleucine, leucine, and phenylalanine with lower tertiles of eGFR in the adjusted models (*p* < 0.01–0.001). The findings hold a promising potential of using plasma isoleucine, leucine, and phenylalanine levels for evaluation of kidney function decline. Future longitudinal studies should investigate the causal association between altered levels of these amino acids and impaired kidney function and also the utility of the former as potential biomarkers for evaluating the risk and early detection of the latter.

## 1. Introduction

Impaired kidney function, usually defined by a reduction in the estimated glomerular filtration rate (eGFR), is a major health problem in both developing and developed countries. Impairment in kidney function has consistently been shown to be an independent risk factor for the development and progression of a wide variety of adverse health outcomes, such as cardiovascular and cerebrovascular disease, anemia and infections, impairment in cognitive function, peripheral artery disease, etc. [1,2,3,4,5,6]. Thus, kidney diseases with kidney function decline pose remarkably high risks for morbidity and mortality with enormous burdens and challenges to the global public healthcare system [7,8]. However, the disease is often not detected until it reaches the advanced stages. An improved understanding of the potential determinants of kidney function decline is of utmost importance, which might help to improve our understanding of the relevant pathophysiology and also to undertake specific measures in the prevention and management of it.

In recent years, a number of studies suggested the important roles of specific plasma-free amino acids (PFAAs) in the development and progression of a wide variety of human diseases [9,10]. Additionally, there is an increasing number of research works assessing the utility of PFAAs as potential disease biomarkers [10,11,12,13]. Among the investigated PFAAs, branched-chain amino acids/BCAAs (isoleucine/ile, leucine/leu, and valine/val) and aromatic amino acids/AAAs (phenylalanine/phe, tyrosine/tyr, and tryptophane/trp) have been suggested as important metabolic regulators involved in the pathophysiology of different disease states like hypertension, metabolic syndrome, insulin resistance, type 2 diabetes, etc. [9,14,15,16]. On the other hand, these adverse health conditions are associated with the development of chronic kidney disease (CKD) with impaired kidney function [17]. The existing literature shows that a few studies investigated the circulatory levels of BCAAs or AAAs in chronic kidney disease, and they suggested that BCAA and AAA metabolic disorders may be related to the latter [18,19].

Based on the findings of the available published studies, it is reasonable to postulate that a close link might exist between the altered circulatory levels of BCAAs and AAAs with impaired functions of the kidneys [20]. Hence, revealing the potential association of circulatory levels of BCAAs and AAAs with eGFR decline might help to improve our understanding of the underlying disease pathophysiology and contribute to the assessment of the severity of impaired kidney function. More importantly, such alterations in BCAAs and AAAs might be useful for early identification of persons at high risk of kidney function decline, which would greatly affect the morbidity, mortality, and quality of life among probable patients with impairments in kidney function. However, relevant previous studies investigating the roles of BCAAs and AAAs in kidney function decline mainly performed a comparison of the levels of BCAAs and/or AAAs between patients with CKD and control subjects [18,19]. Moreover, those studies did not adjust the observed results for the potential confounding variables as such patients with CKD may have comorbidities like hypertension and diabetes mellitus, and the latter might confound the relevant findings. Moreover, the findings of the available studies regarding the roles of BCAAs and AAAs in kidney function decline are inconsistent as those studies reported a significant increase, no change, or decrease in the circulating concentrations of specific amino acids among CKD patients [10,19,20,21,22]. Furthermore, there is a lack of studies that focused on the association of BCAAs and AAAs with early declines in kidney function. Determination of useful biomarkers capable of earlier detection of kidney function decline would allow for necessary interventions that might prevent the progression of such impaired kidney function and associated complications. Considering all these—as the initial step in identifying useful biomarkers of early kidney function decline—it seems important to understand the potential existence of any association between the circulatory levels of BCAAs and AAAs with eGFR decline, in a relatively large healthy population.

Accordingly, one purpose of this cross-sectional study was to investigate the possible differences in the concentrations of BCAAs and AAAs according to the tertiles of eGFR, amongst apparently healthy subjects. The second and the main purpose was to explore and clarify the possible existence of any relationships of the altered levels of individual BCAAs and AAAs with eGFR, after adjustments for potential confounders. We hypothesized that specific patterns of concentrations of BCAAs and/or AAAs would be associated with progressive reduction in eGFR.

## 2. Materials and Methods

### 2.1. Study Design, Population, and Data Collection

**Figure 1:**
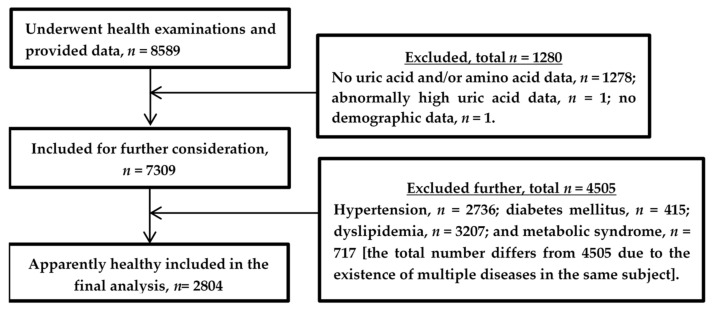
Flowchart of current study population.

In the current research work, we performed secondary analyses of the data collected for a cross-sectional study conducted in Shimane Prefecture, Japan and presented elsewhere [23,24]. Figure 1 depicts the flowchart of the study participants included in this study. Briefly, the final study population comprised a total of 2804 adult individuals who underwent their annual health check-up at different health examination centers in Shimane. The annual health check-up included physical examinations and clinical and other laboratory tests. Additionally, data were collected on their personal and medical history using a self-administered questionnaire. The subjects of this study were free from any known diseases including renal impairment or cancer. Currently, none of them were taking any medications. For exclusion of subjects with lifestyle-related diseases, we used the following criteria: (a) diabetes mellitus, defined as fasting plasma glucose/FPG level of ≥126 mg/dL, hemoglobin A1c/HbA1c level of ≥6.5%, and/or the use of medication for diabetes mellitus; (b) hypertension, defined as systolic blood pressure/SBP of ≥140 mmHg or diastolic blood pressure/DBP of ≥90 mmHg, and/or the use of any antihypertensive medications; (c) dyslipidemia, defined as low-density lipoprotein cholesterol/LDLC level of ≥140 mg/dL, high-density lipoprotein cholesterol/HDLC level of <40 mg/dL, triglyceride/TG  level of ≥150 mg/dL, and/or the use of medication for dyslipidemia; (d) metabolic syndrome, defined according to the Japanese criteria used for diagnosis of the syndrome (waist circumference ≥ 85 cm in males and ≥90 cm in females plus at least two of the following three components: HDLC < 40 mg/dL, TG ≥ 150 mg/dL, and/or the use of medication for dyslipidemia; FPG ≥ 110 mg/dL and/or the use of medication for diabetes mellitus; and blood pressure ≥ 130/85 mmHg and/or the use of antihypertensive medication) [14].

### 2.2. Collection and Storage of Blood Samples

Venous blood samples were drawn from the cubital vein of a seated subject after an 8-h overnight fast. Five mL of blood samples were collected into tubes that contained ethylenediaminetetraacetic acid (EDTA; Terumo, Tokyo, Japan). The tubes were put on ice immediately and kept there for a period of about 15 min. Next, the tubes were incubated at 4 °C by centrifugation at 3000 rpm for 15 min. This was followed by separation of the plasma into tubes, which were stored at −80 °C for a period of 2 weeks to 2 months until the desired analysis for amino acids was performed.

### 2.3. Measurement of Amino Acids and Other Laboratory Variables

In the current study, we measured the absolute concentrations (in μmol/L) of BCAAs and AAAs following the protocol as described elsewhere [25,26]. Briefly, before the measurements of amino acids, the plasma samples were deproteinized using acetonitrile at a final concentration of 80%. Precolumn derivatization was performed with 3-aminopyridyl-N-hydroxysuccinimidyl-carbamate (APDSTAG™, Wako Pure Chemical Industries, Ltd., Osaka, Japan). After this, the concentrations of BCAAs and AAAs were measured by high-performance liquid chromatography–electrospray ionization–mass spectrometry (HPLC–ESI–MS), which allows such measurements with high accuracy. HPLC analysis was conducted on a Shim-pack UF-Amino column (C18 reverse-phase column, Shimadzu Corporation, Kyoto, Japan).

FPG and HbA1c were measured using the hexokinase method and latex agglutination immunoassay, respectively. Measurements of the concentrations of HDLC, LDLC, and TG in the serum were performed enzymatically. Plasma UA was measured using the uricase-HMMPS method by L-type UA.M kit (Wako Pure Chemical Industries, Ltd., Osaka, Japan). The serum creatinine level was determined by using the creatininase-HMMPS method following the manufacturer’s instructions (Wako Pure Chemical Industries, Osaka, Japan).

### 2.4. Calculation of eGFR

For each subject included in this study, the eGFR was calculated using individual serum creatinine data and applying the following equation recommended for the Japanese population by the Japanese Society of Nephrology: eGFR (mL/min/1.73 m^2^) = 194 × serum creatinine (−1.094) × age (−0.287) × 0.739 (if female) [27].

### 2.5. Statistical Analyses

The continuous variables of this study did not show the normal distribution as evaluated by the Kolmogorov–Smirnov and Shapiro–Wilk tests. Therefore, non-parametric tests were employed as applicable. For the continuous demographic and clinical variables, the differences between the groups were assessed by the Kruskal–Wallis test and the Mann–Whitney U-test with Bonferroni corrections for multiple comparisons as necessary and for the categorical variable by the Chi-square (χ2) test. The continuous variables of this study have been presented as median and interquartile range (IQR), and the categorical variable has been presented as a number and a percentage.

We categorized the subjects of this study into three tertiles (tertile 1, lowest; tertile 2, middle; and tertile 3, highest) based on the tertile values of the eGFR level of the current study population. The corresponding cutoffs were <78.17 mL/min/1.73 m^2^, 78.17–92.03 mL/min/1.73 m^2^, and >92.03 mL/min/1.73 m^2^ for tertiles 1, 2, and 3, respectively. Spearman’s rank correlation analysis was performed between the concentrations of BCAAs and AAAs separately in all tertile categories.

In this study, a decline in eGFR was defined as a decreasing trend from the higher to the lower tertiles of eGFR. We explored the association between eGFR decline and individual amino acids by multinomial logistic regression analyses (highest tertile or tertile 3 of eGFR as the reference category) with adjustments for potential confounders. For the analyses, the values of BCAAs and AAAs were scaled to multiples of 1 IQR calculated separately for males and females. We adjusted the logistic regression models for only demographic variables (1st model), demographic and clinical variables excluding UA (2nd model), and demographic and clinical variables including UA (3rd model). From the logistic regression analyses, we obtained the odds ratios (OR) for individual amino acids with corresponding 95% confidence intervals (CI) and *p*-values. The statistical analyses of the data were performed with the software package SPSS version 22 for Windows (SPSS Inc., Chicago, IL, USA). All statistical tests were considered as two-tailed, and the significance level was set at *p* < 0.05.

## 3. Results

The data collected from a total of 2804 healthy subjects (1191 men, 1613 women) apparently free from any diseases and not taking any medications were included in the final analysis of this study (Figure 1).

### 3.1. Demographic and Clinical Characteristics of the Study Subjects

The demographic and clinical characteristics of the current study subjects, according to the tertiles of eGFR, are presented in Table 1. The group differences were significant for all these variables (Kruskal–Wallis test, *p* < 0.05–0.001). Additionally, the group difference for the level of eGFR between the tertile categories was highly significant (Kruskal–Wallis test, *p* < 0.001).

As observed, all the tertile categories of eGFR included more females with a significant difference in the sex distribution across the tertiles (χ2 test, *p* < 0.005). Compared to the subjects in tertile 3 (i.e., with highest eGFR), those in tertiles 1 and 2 (i.e., with lower eGFR) were older with higher BMI (Table 1). Additionally, in comparison with the subjects in tertile 3, subjects in the lower tertiles had significantly elevated levels of FPG, HbA1c, LDLC, TG, SBP, and DBP (Mann–Whitney *U*-test, *p* < 0.001), and subjects in the lowest tertile had significantly lower levels of HDLC (Mann–Whitney U-test, *p* < 0.05).

### 3.2. Differences in the Concentrations of BCAAs and AAAs between eGFR Tertiles

Table 2 shows the median and IQR values for the concentrations of individual BCAAs (ile, leu, and val), and AAAs (phe, trp, and tyr), according to the tertiles of eGFR. The analyses revealed that all BCAAs and AAAs had significant group differences, except for trp, between the tertile categories (Kruskal–Wallis test, *p* < 0.001). There was a progressive increase in the concentrations of all BCAAs and AAAs from the higher to the lower tertiles

### 3.3. Correlation between BCAAs and AAAs

The correlations between the measured concentration of BCAAs and AAAs in different tertiles were examined with Spearman’s rank correlation analyses. As evident in Table 3, plasma concentrations of BCAAs and AAAs showed significant moderate correlations (positive) with each other in all tertile categories of eGFR (*r* = 0.43–0.56 for tertile 1, and 0.41–0.59 for both tertile 2 and tertile 3; *p* < 0.001).

### 3.4. Association of BCAAs and AAAs with eGFR

We investigated the potential association between altered levels of BCAAS and AAAs with eGFR after adjustments for potential confounders by multinomial logistic regression analyses, considering the corresponding highest tertile of eGFR as the reference category (Table 4). As revealed, there was a consistent trend of associations of ile, leu, and phe with eGFR in all adjusted models.

As the current analysis shows, among the BCAAs, ile and leu showed significant associations with eGFR in the lowest tertile category in all adjusted models (ORs of 1.21–1.29 and 1.24–1.42, 95% CIs of 1.04–1.13 and 1.06–1.23 (lower), and 1.41–1.49 and 1.46–1.64 (upper) for ile and leu, respectively; *p* < 0.001–0.05).

Among the AAAs, the analysis revealed a significant association with eGFR, only for phe, in both lower tertile categories of all adjusted models (ORs of 1.21–1.24 and 1.53–1.65, 95% CIs of 1.05–1.08 and 1.31–1.38 (lower), and 1.39–1.43 and 1.78–1.92 (upper) for tertiles 2 and 1, respectively; *p* < 0.001–0.01).

## 4. Discussion

The kidneys play crucial roles in the homeostasis of circulating and tissue amino acid pools through synthesis and release, degradation, filtration and reabsorption, and urinary excretion of the latter [28]. In humans, any alterations in the circulating levels of BCAAs and AAAs related to kidney function impairment might have profound effects on various biological mechanisms and physiological functions including cell signaling, gene expression, and neuroendocrine function [29]. To clarify the association between circulatory levels of BCAAs and AAAs with kidney function decline, in this study, we investigated and characterized these PFAAs according to the tertiles of eGFR in a large group of healthy people. Our results indicate that alterations in BCAA and AAA levels might be closely associated with a decline in eGFR.

As reported in the literature, the prevalence of decreased kidney function becomes higher with increased age in both men and women and in persons with diseases like diabetes or hypertension compared to those without such disease states [30,31]. Therefore, our observed findings of significantly higher values of different demographic and clinical variables among subjects with kidney function decline are in line with the current literature.

Published epidemiologic and experimental evidence suggests a clear relationship of elevated circulatory UA levels with reduced eGFR. It has been proposed that the role of high circulatory UA (known as hyperuricemia) might be causal in the development of kidney function impairment [32,33]. Therefore, our finding of a significantly higher concentration of UA among the subjects with reduced eGFR corresponds to the existing literature. Considering this potential role of elevated levels of UA in reduced eGFR, we adjusted our results of logistic regression analysis for UA along with other demographic and clinical variables investigated in the current study.

In this study, compared to the corresponding highest tertile, the concentrations of all BCAAs and AAAs (except trp) showed a significant increase in the lower tertiles of eGFR. In the human body, there is a consistent uptake of the BCAAs by the kidneys [34]. Therefore, a significant increase in BCAA levels in the lower tertiles of eGFR might reflect their reduced uptake by the kidneys with declines in eGFR. On the other hand, the kidneys take up phe from the bloodstream and convert it to tyr with subsequent release into renal veins [35]. Therefore, it might be possible that impaired kidney function causes diminished renal uptake of phe, which may lead to a higher level of it and a concomitant lower level of tyr in the plasma. On the other hand, the reason for a significantly higher level of tyr in the lower tertiles of eGFR observed in this study remains unclear. However, it might reflect the fact that the subjects in the current study were apparently healthy with preserved kidney functions and capable of converting increased amounts of phe to tyr. Furthermore, it has been suggested that BCAAs and AAAs compete for transport into mammalian cells by the common large neutral amino acid transporter, LAT1 [36,37]. Therefore, we postulate that an increase in AAA levels among the subjects in the lower tertiles of eGFR might have been partially driven by the higher levels of BCAAs among them. This is further supported by our observation of significant positive correlations between BCAAs and AAAs in all tertile categories, which reflects the possible existence of a close link between the circulating levels of BCAAs and AAAs as suggested in a previous work [24].

Our findings on the alterations in BCAA and AAA levels according to eGFR tertiles cannot be directly compared to those of others as in the latter studies; the levels of PFAAs had been generally compared between patients with CKD and control subjects [18,19,20,38]. The situation has been further complicated by the relevant published data that show discrepant and/or inconsistent results. For example, similar to our findings, Ceballos et al. (1990) reported an increasing trend (not significant) in the plasma concentrations of all BCAAs among patients with a mild degree of renal failure (Ccr > 25 mL/mn), compared to healthy controls [22]. In contrast, in a study by Suliman et al. (2005), plasma concentrations of leu and val were significantly lower in CKD patients compared with the control subjects; however, plasma levels of ile showed a slightly higher value (not significant) in the patients [20]. Similarly, Kumar et al. (2012) also observed a significant decrease in the plasma concentrations of leu and val in CKD patients when compared with the control group [18]. On the other hand, among the AAAs, the plasma level of phe showed a significant increase among the patients with CKD in comparison with controls [38,39]. This is consistent with our finding of a significant elevation in plasma phe among subjects in the lower tertiles of eGFR. Conversely, Li et al. (2011) and Suliman et al. (2005) did not observe any significant difference in the circulating level of phe among CKD patients compared with control subjects [19,20]. Among the other two AAAs, in our study, we observed a significant increase in tyr level only in the lower tertiles of eGFR. In contrast, Li et al. (2011) and Suliman et al. (2005) revealed that the blood concentrations of tyr and trp were significantly lower in CKD patients compared with the control subjects [19,20]. All these findings and observations demonstrate a profound lack of consensus among the researchers regarding the patterns of circulating levels of BCAAs and AAAs among subjects with reduced kidney function. In our study, we compared PFAAs between the tertile categories of eGFR in an apparently healthy population and with preserved kidney functions. Conversely, other studies included patient populations with CKD and varying degrees of disease severity. All these might have played a role in the observed differences for the levels of PFAAs observed among the study populations investigated in the above-mentioned research work.

In this study, we confirmed the associations between altered levels of BCAAs and AAAs with the eGFR decline after adjustments for potential confounders. Among the BCAAs and AAAs, the current results for all adjusted logistic regression analyses revealed a consistent trend in the associations between ile, leu, and phe with a decline in eGFR and demonstrated a progressive increase in the corresponding ORs for lower tertiles in all models. Overall, the association was significant in the lowest tertile category for ile, leu, and phe. For phe, a significant association was also observed in the middle/second tertile category of eGFR. These findings reflect the existence of a possible close link between altered levels of ile, leu, and phe with a decline in eGFR. There is a lack of similar published studies with investigation of the possible associations between changes in BCAA and AAA levels and early impairments in kidney function. This severely limits the scope for comparisons of our findings with those of others. However, in an epidemiological study with eight years follow-up of amino acid metabolites associated with CKD in the general population, the authors revealed that among other amino acids, ile, leu, and phe were negatively correlated with eGFR [39]. Furthermore, in that study, ile, leu, and phe demonstrated significant positive associations with CKD after adjustments for age, sex, body mass index, smoking status, drinking status, systolic blood pressure, HbA1C, and hs-CRP in the multivariate logistic regression analyses [39].

From our study design, it is not possible to discern whether the changes in the BCAA and AAA levels were a cause or a consequence of probable changes in kidney function with a decline in eGFR, or vice versa. Additionally, the exact mechanisms underlying the observed association between the BCAA and AAA levels and reduced eGFR cannot be explained from our findings due to the current research design. However, considering our findings and those of others, it is obvious that a decline in eGFR might be closely related with alterations in plasma levels of ile, leu, and phe. Moreover, our findings suggest the possibility of using plasma ile, leu, and phe as potential biomarkers for the evaluation of reduced kidney function.

The current study findings need to be interpreted in light of several potential limitations. First, for this study, data on the background information such as diet, physical exercise, alcohol drinking, tobacco smoking, etc. were not available. However, we firmly believe that any effects of such factors on the current study results should be minimal as all the subjects included in this study had similar socio-demographic characteristics. Second, the results were not stratified by the variable of “sex.” However, we believe this did not influence our study results as we used different equations for calculating eGFR for men and women, respectively. Moreover, the results of logistic regression analyses were adjusted for the variable “sex’” along with other potential confounders. Third, despite high statistical significance, we revealed relatively low ORs for the association between specific PFAAs with eGFR. We believe that inclusion of healthy subjects in this study might be an important contributor to such observed ORs in the logistic regression models. Fourth, measurements of BCAAs and AAAs in an apparently healthy population according to the tertiles of eGFR may not reflect the exact changes observed under the natural course of the disease with kidney function decline. However, we believe that the trends in the eGFR level and its relations with specific PFAAS, observed by us, are likely to reflect the true changes in these variables among subjects with impaired kidney function. Fifth, the generalization of the findings of this study is somewhat limited as this study was conducted amongst the Japanese population. Lastly, the cross-sectional design of this study does not allow us to speculate on any causality or temporality for the associations observed in this study between altered BCAA and AAA levels and declines in eGFR.

## 5. Conclusions

In this study, high concentrations of ile, leu, and phe were significantly related to eGFR decline. We suggest that altered concentrations of these PFAAs might be closely associated with kidney function and may be useful for early identification of declines in kidney function. Future longitudinal studies should investigate the utility of plasma ile, leu, and phe as potential biomarkers for evaluating the risk of declines in kidney function and its early detection. Furthermore, the causal associations between alterations in the plasma levels of BCAAs and AAAs and decline in kidney function and their possible interactions need to be elucidated, which might provide new insights into the pathophysiology of the discussed disease state and the development of intervention strategies directed towards its prevention and management.

## Figures and Tables

**Table 1:** Demographic and clinical characteristics of study subjects. Values are expressed as median and interquartile range (IQR) for continuous variables and as a number and a percent (total or within tertiles) for the categorical variable.

Variable	Total		Tertile 1		Tertile 2		Tertile 3		P1	P2	P3
	(*n* = 2804)		(*n* = 935)		(*n* = 933)		(*n* = 936)				
	Median	IQR	Median	IQR	Median	IQR	Median	IQR			
	or *n*	or %	or *n*	or %	or *n*	or %	or *n*	or %			
Age (years)	44.0	26.0	59.0	23.0	44.0	22.0	34.0	15.0	<0.001	<0.001	<0.001
Sex											
Male	1191	42.5	413	44.2	427	45.8	351	29.5	<0.005	<0.01	<0.001
Female	1613	57.5	522	55.8	506	54.2	585	36.3			
BMI (kg/m^2^)	21.2	3.7	21.5	3.7	21.4	3.8	20.8	3.6	<0.001	<0.001	<0.005
FPG (mg/dL)	91	10	92	10	91	10	90	9	<0.001	<0.001	<0.005
HbA1c (%)	5.5	0.5	5.6	0.4	5.5	0.5	5.4	0.4	<0.001	<0.001	<0.001
HDLC (mg/dL)	68	22	67	22	69	22	68	20	<0.05	<0.05	1.000
LDLC (mg/dL)	107	31	112	28	108	31	102	30	<0.001	<0.001	<0.001
SBP (mmHg)	118	17	120	17	119	16	116	15	<0.001	<0.001	<0.001
DBP (mmHg)	73	13	74	12	74	12.5	71	13	<0.001	<0.001	<0.001
TG (mg/dL)	64	38	71	36	64	38	58	35	<0.001	<0.001	<0.001
UA (mg/dL)	4.6	1.8	4.9	1.8	4.6	1.9	4.3	1.7	<0.001	<0.001	<0.001
Waist (cm)	76	11.5	78	11.5	76.5	10.5	74	11	<0.001	<0.001	<0.001
eGFR (mL/min/1.73 m^2^)	84.9	21.2	70.9	9.7	84.9	6.6	101.7	13.5	<0.001	<0.001	<0.001

BMI, body mass index; FPG, fasting plasma glucose; HbA1c, haemoglobin A1c; HDLC, high-density lipoprotein cholesterol; LDLC, low-density lipoprotein cholesterol; SBP, systolic blood pressure; DBP, diastolic blood pressure; TG, triglyceride; UA, uric acid; waist, waist circumference. Missing data: FPG, *n* = 115, 59, and 26 for tertiles 1, 2, and 3, respectively; waist, *n* = 49, 28, and 10 for tertiles 1, 2, and 3, respectively. P1 indicates the *p*-values for two-tailed Kruskal–Wallis test for k-independent samples. P2 and P3 indicate the *p*-values for two-tailed Mann–Whitney *U*-test for 2-independent samples: tertile 3/highest tertile versus tertile 1/lowest tertile and tertile 3/highest tertile versus tertile 2/middle tertile, respectively, with adjustments by Bonferroni corrections for multiple corrections.

**Table 2:** BCAA and AAA concentrations (μmol/L) in the study populations. Values are shown as median and interquartile range (IQR).

	Tertile 1		Tertile 2		Tertile 3		P1	P2	P3
Amino	(*n* = 935)		(*n* = 937)		(*n* = 932)				
Acids	Median	IQR	Median	IQR	Median	IQR			
Ile	52.7	16.6	51.5	16.7	50.8	14.6	<0.005	<0.001	0.128
Leu	105.1	28.9	103.1	30.0	99.2	29.0	<0.001	<0.001	<0.005
Val	194.6	52.8	191.6	49.7	187.0	49.0	<0.001	<0.001	<0.05
Phe	56.4	10.3	53.7	9.0	51.3	9.0	<0.001	<0.001	<0.001
Trp	51.6	11.7	51.6	12.1	50.6	12.0	0.059	0.074	0.084
Tyr	59.4	14.8	57.4	14.6	54.3	13.3	<0.001	<0.001	<0.001

Ile, isoleucine; leu, leucine; val, valine; phe, phenylalanine; trp, tryptophan; tyr, tyrosine. P1 indicates the *p*-values for two-tailed Kruskal–Wallis test for k-independent samples. P2 and P3 indicate the *p*-values for two-tailed Mann–Whitney U-test for 2-independent samples: tertile 3/highest tertile versus tertile 1/lowest tertile and tertile 3/highest tertile versus tertile 2/middle tertile, respectively, with adjustments by Bonferroni corrections for multiple comparisons.

**Table 3:** Correlation coefficients between the concentrations of BCAAs and AAAs for healthy control subjects and subjects with hypertension derived by Spearman rank correlation analysis.

Amino	Tertile 1			Tertile 2			Tertile 3		
Acids	Phe	Trp	Tyr	Phe	Trp	Tyr	Phe	Trp	Tyr
Ile	0.49 *	0.51 *	0.43 *	0.48 *	0.53 *	0.41 *	0.48 *	0.51 *	0.41 *
Leu	0.56 *	0.54 *	0.44 *	0.59 *	0.57 *	0.47 *	0.59 *	0.58 *	0.47 *
Val	0.51 *	0.54 *	0.44 *	0.53 *	0.55 *	0.49 *	0.51 *	0.56 *	0.45 *

* All *p*-values: <0.001.

**Table 4:** Logistic regression analysis for association between BCAAs and AAAs with tertiles of eGFR.

	eGFR	Model 1 ^a^				Model 2 ^b^				Model 3 ^c^			
Amino	Tertile	OR	95% CI		*p*-Value	OR	95% CI		*p*-Value	OR	95% CI		*p*-Value
Acids	Categories		Lower	Upper			Lower	Upper			Lower	Upper	
Ile	Tertile 3	Ref				Ref				Ref			
	Tertile 2	1.05	0.92	1.19	0.461	1.10	0.97	1.26	0.152	1.08	0.94	1.24	0.271
	Tertile 1	1.29	1.13	1.47	<0.001	1.29	1.11	1.49	<0.005	1.21	1.04	1.41	<0.05
Leu	Tertile 3	Ref				Ref				Ref			
	Tertile 2	1.12	0.98	1.28	0.103	1.17	1.02	1.35	0.027	1.12	0.97	1.29	0.124
	Tertile 1	1.42	1.23	1.64	<0.001	1.38	1.18	1.62	<0.001	1.24	1.06	1.46	<0.01
Val	Tertile 3	Ref				Ref				Ref			
	Tertile 2	0.99	0.87	1.13	0.933	1.06	0.92	1.21	0.451	1.01	0.87	1.16	0.948
	Tertile 1	1.13	0.98	1.30	0.102	1.10	0.94	1.28	0.242	0.98	0.83	1.15	0.782
Phe	Tertile 3	Ref				Ref				Ref			
	Tertile 2	1.22	1.06	1.39	<0.005	1.24	1.08	1.43	<0.005	1.21	1.05	1.39	<0.01
	Tertile 1	1.59	1.38	1.83	<0.001	1.65	1.41	1.92	<0.001	1.53	1.31	1.78	<0.001
Trp	Tertile 3	Ref				Ref				Ref			
	Tertile 2	0.95	0.84	1.07	0.399	0.93	0.82	1.06	0.265	0.92	0.81	1.04	0.165
	Tertile 1	0.93	0.82	1.06	0.293	0.89	0.78	1.03	0.121	0.87	0.76	1.01	0.064
Tyr	Tertile 3	Ref				Ref				Ref			
	Tertile 2	0.98	0.86	1.12	0.775	1.01	0.88	1.17	0.897	0.97	0.84	1.12	0.681
	Tertile 1	0.98	0.86	1.12	0.775	0.98	0.83	1.15	0.776	0.89	0.76	1.05	0.169

^a^ Adjusted for age, sex, and BMI; ^b^ adjusted for variables in model 1 plus FPG, HbA1c, HDLC, LDLC, SBP, DBP, TG, and waist circumference; ^c^ adjusted for variables in model 2 plus UA. OR, odds ratio; CI, confidence interval; Ref, reference category.

## Data Availability

The data presented in this study are available from the corresponding author upon reasonable request.
